# Genetics and Functional Genomics of Spondyloarthritis

**DOI:** 10.3389/fimmu.2018.02933

**Published:** 2018-12-18

**Authors:** Félicie Costantino, Maxime Breban, Henri-Jean Garchon

**Affiliations:** ^1^UMR 1173 INSERM/Versailles Saint-Quentin-en-Yvelines University, Montigny le Bretonneux, France; ^2^Rheumatology Division Ambroise Paré Hospital (AP-HP), Boulogne-Billancourt, France; ^3^Genetics Division Ambroise Paré Hospital (AP-HP), Boulogne-Billancourt, France

**Keywords:** spondyloarthritis, ankylosing spondylitis, genetics, genomics, GWAS, family-based, next-generation sequencing

## Abstract

Spondyloarthritis (SpA) is a chronic inflammatory disorder with high heritability but with complex genetics. It encompasses several entities that share common clinical features. Most of the genetic studies in SpA have been restricted to ankylosing spondylitis (AS), the prototypical form of SpA. However, there is growing evidence of shared genetic background between all the SpA subtypes and also with some other immune-mediated diseases. The most important part of SpA heritability comes from the HLA-B27 allele in the major histocompatibility complex (MHC) that explains around 25% of the attributable heredity. Several other loci outside of the MHC have been shown to be involved in the disease. However, all these non-MHC loci explain only a small additional fraction of disease predisposition. Thus, a substantial fraction of SpA genetic basis remains poorly understood. Gene expression profiling is a complementary approach to elucidate the underlying mechanisms and pathways that drive the disease. Several expression profiling studies have been undertaken in SpA. However, results have been quite disappointing with little overlap between the studies largely due to the small sample sizes, resulting in limited power to discover small effects. In this review, we summarize current knowledge on genetic findings concerning SpA and we describe strategic approaches for identification of additional variants, with a focus on rare variants in familial forms. We also provide an overview of gene expression studies in SpA and discuss the possibilities offered by high-throughput RNA sequencing technologies, in particular in sorted cells. Finally, issues in establishing molecular mechanisms underlying genetic association hits and potential translational applications will be addressed.

## Introduction

Spondyloarthritis (SpA) is one of the most common forms of chronic inflammatory rheumatism with an estimated prevalence of 0.54% in the Western adult population (1). It is characterized by inflammation of the spine, frequently extending to the peripheral joints. Extra-articular inflammatory manifestations such as psoriasis, uveitis or inflammatory bowel diseases (IBD) are also common. Depending on its clinical and radiological presentation, several subsets of the disease have been defined: ankylosing spondylitis (AS), psoriatic arthritis, arthritis associated with IBD, reactive arthritis, and undifferentiated SpA.

The etiology of SpA is multifactorial with a strong genetic component dominated by the HLA-B27 allele. In recent years, numerous other genetic factors of susceptibility have been uncovered, most of them through genome-wide association studies (GWAS). However, these factors explain only a small fraction of disease predisposition.

Although we currently do not have a comprehensive picture of the genetic background of SpA, GWAS hits have enhanced our understanding of SpA pathogenesis, notably by highlighting several major pathways involved in the disease. Gene expression profiling can provide additional information on SpA genetic architecture with a more functional point of view. The new possibilities offered by high-throughput RNA sequencing technologies, in particular in sorted cells, are very promising. Functional genomics will also help to establish molecular mechanisms underlying genetic associations. The ultimate objective is to drive translational advances leading to a more effective diagnosis and treatment of disease.

## Genetics of Spondyloarthritis

There is a growing understanding of spondyloarthritis genetic background, with both MHC and non-MHC loci already identified. However, a large fraction of the disease heritability remains to be elucidated.

### Genetic Epidemiology of SpA

#### A Strong Genetic Background

Familial aggregation of SpA cases has been recognized for a long time and was the first evidence that genetic factors may predispose to the disease (2). The recurrence risk ratio of the sibling or first-degree relative of a patient was estimated to 80 in AS and 40 in SpA as a whole (3, 4). It was initially unclear whether the co-familiality was due to shared environment or genetic factors. However, the discovery of the strong association between HLA-B27 allele and SpA and studies of disease recurrence in twins are in favor of a strong genetic component in SpA and heritability was estimated at 90% (5–7).

Before moving on to the results of genetic studies in SpA, it seems important to discuss disease phenotype. Indeed, a reliable phenotype definition is essential for interpreting the meaning and validity of genetic studies. Restriction to the well-defined AS phenotype, requiring an advanced radiological sacroiliitis, increases the genetic homogeneity of the studied cohorts, and thus improves the statistical power of the analyses (8). However, other subtypes of SpA have been found among family members of patients with AS suggesting shared genetic factors (4, 9). Taking these cases into considerations should provide a comprehensive picture of the genetic factors involved in SpA, especially in family-based studies.

#### HLA-B27: Not the Only One!

The association between HLA-B27 and SpA was first reported in the early 1970s (10, 11). This is one of the strongest associations between a common disease and an HLA antigen with a relative risk of SpA in HLA-B27 positive individuals nearing 40 (12). However, epidemiological data suggested that susceptibility to SpA is affected by other genes. Indeed, HLA-B27 positive relatives of AS patients have a recurrence risk of the disease 16 times greater than HLA-B27 positive individuals from the general population (13). Twin studies are also in favor of a non-B27 genetic contribution to susceptibility to AS, as the greater concordance rate observed in monozygotic twins compared to dizygotic twins persists in B27 positive twin pairs (5). An oligogenic model of transmission has been suggested based on the observed pattern of recurrence in families (3).

### Genetic Factors of Susceptibility to SpA

#### Major Histocompatibility Complex

The MHC region plays a major role in SpA with a contribution recently estimated to 20.5% of the heritability (14). HLA-B27 is by far the main factor of SpA susceptibility but there is also strong evidence for additional susceptibility alleles in the MHC (15).

##### HLA-B27

The association between HLA-B27 and AS was first reported in the early 70's and then confirmed in other subtypes of the disease (10, 11, 16). HLA-B^*^27 is highly polymorphic with several hundreds of subtypes already identified (17). Association with SpA has been well established for the most frequent subtypes, i.e., HLA-B^*^27:05, the ancestral allele from which other subtypes have derived, B^*^27:02, B^*^27:04, and B^*^27:07 (18). Two other subtypes, B^*^27:06 (a common subtype in South-East Asia) and B^*^27:09 (a rare subtype found in Sardinia) are of particular interest since they were reported as less or not associated with SpA (19, 20). The other subtypes are too rare to determine whether they are associated with SpA or not.

There is no doubt that the HLA-B27 is the main genetic factor predisposing to SpA. However, the mechanism of such association remains as yet unproven and several distinct theories have been proposed to explain it (16, 21). Although detailing these theories is beyond the scope of this review, we will briefly describe the three main ones. The oldest theory involves specific antigenic presentation leading to autoimmunity through molecular mimicry between pathogen and self-derived epitopes (22). The two others are more recent and postulate a direct role of HLA-B27 as a pro-inflammatory actor which stimulates the Th17 axis through misfolding of the HLA-B27 heavy chain (23) or formation of heavy chain homodimers at the cell surface (24).

##### Other HLA alleles

Identification of other MHC alleles associated with SpA is challenging because of the overwhelming influence of HLA-B27 itself. While non-B27 HLA associations have been reported, notably with HLA-B^*^40 (25, 26) and HLA-A^*^02 (27), most have not been definitive or replicated in independent studies. Using MHC data from Immunochip microarray, Cortes et al. have identified several other HLA alleles associated with AS (15). This large study including 9,069 cases and 13,578 controls of European descent showed that in addition to effects due to B27 alleles, several other HLA-B increased disease susceptibility (B^*^13:02, B^*^40:01, B^*^40:02, B^*^47:01, B^*^51:01) whereas other alleles were protective (B^*^07:02, B^*^57:01). After controlling for the associated HLA-B alleles, several independent associations were also identified with variants in the HLA-A, HLA-DPB1, and HLA-DRB1 loci.

##### Other MHC genes

Involvement of other non-HLA MHC genes (e.g., *MICA, TNF, TAP1, TAP2*, and *LMP2*) has also been suggested (28). However, linkage disequilibrium with HLA-B27 makes these associations difficult to interpret. Two recent studies investigating the participation of MHC class I polypeptide-related sequence A (MICA)^*^007:01 allele to AS susceptibility illustrate these difficulties. Indeed Zhou et al. reported in 2014 a strong association of this allele with AS in cohorts from North America and China (29) but this was not confirmed in a recent well-powered study in a large cohort of European ancestry (30).

#### Outside the MHC

No less than 30 years elapsed between the discovery of the association between HLA-B27 and SpA and the identification of other genetic factors of susceptibility to SpA thanks to the development of genotyping techniques. In the meantime, many candidate gene studies were conducted but most of the associations reported in those studies failed to be confirmed (31). With the development of high-throughput genotyping and analysis methods, it became possible to move from candidate-gene studies to genome-wide (i.e., hypothesis-free) association studies (GWAS).

The first GWAS conducted in AS in 2007 led to the identification of 2 non-MHC susceptibility loci in *ERAP1* and *IL23R* (32). These associations were subsequently confirmed in several independent cohorts (33, 34). Since then, 2 other GWAS were published in European ancestry cohorts allowing the identification of 6 additional loci (35, 36). The only GWAS performed in non-European population included 1,837 cases and 4,231 controls from the Han population (37). Two new susceptibility loci were identified but none was replicated even in large studies; thus there are likely to be false-positives. Table 1 summarizes the design and results of genome-wide studies in AS.

**Table 1:** Genome-wide association studies and Immunochip studies in ankylosing spondylitis.

**Study**	**Year**	**Sample size (case/control)**	**Ethnicity**	**Number of variants**	**Number of genome-wide significant loci**
Burton et al. (32)	2007	922/1,466	Caucasian	15,333	2
Reveille et al. (36)	2010	2,053/5,140	Caucasian	286,662	6
Evans et al. (35)	2011	3,023/8,779	Caucasian	2,223,620	8
Lin et al. (37)	2011	1,837/4,231	Chinese Han	1,356,350	3
Cortes et al. (27)	2013	10,619/15,145	Caucasian	128,935	24
Ellinghaus et al. (14)	2016	52,262/34,213	Caucasian	130,052	113

Important progress has been made using the Immunochip microarray which is not a whole-genome microarray but has dense coverage in immune loci and MHC region. In 2013, the International Genetics of Ankylosing Spondylitis Consortium (IGAS) conducted a large case-control study in AS using this chip and identified 13 new associations (27). A combined analysis of Immunochip data from 5 diseases (AS, Crohn's disease, psoriasis, primary sclerosing cholangitis, and ulcerative colitis) increased the statistical power and thus enabled the identification of 113 AS-associated genome-wide significant variants (of which 17 were new loci) (14).

#### Alternative Approaches to Uncover the Unexplained Heritability

Despite important breakthroughs in genetics, only 27.82% of AS heritability is now explained (including 20.44% contributed by HLA-B27 and 7.38% by the other loci) (14). Several hypothesis have been advanced to account for this unexplained heritability. Among them are large variants (deletions, duplications, and inversions) as opposed to single nucleotide variants, gene-gene and gene-environment interactions and rare variants (38).

##### Large variants

Large variants (large deletions, copy number variations, and inversion) are individually rare but collectively common in the human population (39). The available data suggest that these variants can contribute to a variety of different diseases (40). However, the identification of these large variants is difficult to perform at the whole-genome level. In SpA, only copy number variations have been investigated genome-wide in a single study: Jung et al. identified 227 CNV regions associated with AS but successfully replicated only 4 of them (41).

##### Epistasis

Another hypothesis to explain missing heritability is that some common variants show interactions so that their joint effect is greater than the sum of their individual effects. In SpA, no gene-gene interaction study has been performed at the whole-genome level. In the GWAS published in 2011, however, Evans et al. tested for gene-gene interaction between all the loci known to be associated with AS. The only robust interaction observed was between HLA-B27 and the non-synonymous ERAP1 SNP, rs30187 (35).

##### Rare variants

To date, much of the effort to uncover missing heritability has focused on the possible contribution of rare variants. In SpA, few reports identifying rare variants have been published (summarized in Table 2), some of them with a classical case-control design, the others with a family-based design probably more appropriate when dealing about rare variants.

**Table 2:** Studies investigating rare variants in SpA.

**Study**	**Year**	**Design**	**Ethnicity**	**Sample size**	**Gene**
**CASE-CONTROL**					
Robinson et al.(42)	2016	Whole-genome genotyping	Caucasian	5,040 AS/21,133 controls	CDKAL1
**FAMILY-BASED**					
Uddin et al. (43)	2013	Whole-genome CNV	Caucasian	1 family	UGT2B17
O'Rielly et al. (44)	2015	Whole-exome sequencing	Caucasian	1 family	SEC16A/MAMDC4
Rong et al. (45)	2015	Targeted sequencing	Chinese Han	1 family	IRS1
Tan et al. (46)	2018	Whole-genome linkage + exome sequencing	Chinese Han	1 family	ANKDD1B
Feng et al. (47)	2018	Whole-genome linkage + exome sequencing	Chinese Han	1 family	TREML2

One single case-control study investigated the role of rare variants in AS using the Illumina Exomechip microarray. In addition to covering common coding variants, this chip has extensive low-frequency and rare variants content. The study was conducted in a cohort of 5,040 patients and 21,133 healthy controls of European descent. Only one novel genome-wide significant association was noted at *CDKAL1* (42). Despite the large sample size, this study was underpowered to identify rare variants association. Indeed, the power was estimated to 100% for variants with minor allele frequency (MAF) of 5% but decreased dramatically with the MAF: to 9% for MAF of 1% and close to zero for the median MAF in this study (0.02%).

Before the GWAS era, whole genome scans used families data for linkage analysis. In SpA, three genome-wide linkage studies using micro-satellites were published, two in AS and one in SpA as a whole (48–50). Only two loci besides the MHC reached significance threshold: one on 16q and the other on 9q31-34. One limitation of linkage studies is that they cannot locate disease-associated loci on a fine scale. To try to circumvent this issue, a more recent linkage analysis used a high-density panel of SNPs. A new locus significantly linked with SpA was identified on 13q13 but the disease interval could not be restricted to < 1.4 Mb (51). Thus, linkage analysis can be seen as a preliminary step to highlight regions of interest which can be deep-resequenced.

Several studies combined family-based design and next-generation sequencing (Table 2). Rare variants were identified in *SEC16A, MAMDC4* (44), *IRS1* (45), *UGT2B17* (43), *ANKDD1B* (46), and *TREML2* (47). However, most of the time, these variants were not found in other families and the efforts made to validate them by classical approaches such as case-control study failed (44).

### What Have we Learned From GWAS in SpA?

Despite criticism often made to GWAS that they fail to fully explain the heritability of diseases, their greatest strength is their hypothesis-free and unbiased nature. GWAS hits have uncovered previously unsuspected, yet important, biological mechanisms, and pathways involved in SpA, such as aminopeptidases or IL23/IL17 pathways, with a potential therapeutic impact. Comparison of GWAS hits among immune-mediated diseases also led to the concept of shared genetic background.

#### Pathways Involved in SpA Pathogenesis

##### Aminopeptidases

A major discovery of the GWAS has been the association of the M1-aminopeptidase family with AS. Association was first reported in AS with *ERAP1* (35) and then confirmed in other SpA subtypes (52, 53). Associations with variants in three other genes of the same family (*ERAP2, LNPEP*, and *NPEPPS)* were identified later (27). Identification of causal variants and their functional consequences will be detailed later in this review.

*ERAP1* and *ERAP2* code for enzymes expressed in the endoplasmic reticulum; their main function is to trim peptides to the optimal length for binding to MHC class I molecules (54). This function together with the strong genetic interaction demonstrated between *ERAP1* variants and HLA-B27 pinpointed the disturbed peptide presentation as a key molecular mechanism involved in SpA. As most of the functional data are consistent with deleterious increased expression and function of these enzymes, inhibiting ERAP1 and/or ERAP2 functions could have great therapeutic interest in SpA. Indeed, preliminary *ex vivo* data showed that ERAP1 silencing or inhibition in antigen presenting cells suppressed Th17 expansion (55).

##### IL23R

The second pathway highlighted by GWAS hits was the IL23/Th17 axis. The first association identified was with the IL23 receptor gene with the discovery of a loss of function protective variant (32, 56). Associations have also been identified with other genes on the IL23/Th17 pathway: *IL12B, STAT3, CARD9, JAK2, TYK2, IL6R*, and *IL27* (14, 30, 35, 36, 57).

IL-23 is a pro-inflammatory cytokine essential for the differentiation of Th17 lymphocytes, a subtype of T lymphocytes involved in chronic inflammation (58). There is strong evidence linking the IL23/Th17 pathway to SpA. In particular, Sherlock et al. have demonstrated that IL-23 overexpression is sufficient to induce most of the SpA features in the mouse (59). Secukinumab, a fully human monoclonal antibody that binds and neutralizes IL-17A is now approved for AS treatment (60) and several IL-17 targeting drugs are in trials at the moment. Interestingly, ustekinumab, a monoclonal antibody that binds the IL-12/IL-23 shared p40 subunit has recently failed to demonstrate efficacy in AS suggesting differential effects of targeting IL-23 or IL-17 (61).

#### Immune-Mediated Inflammatory Diseases: A Shared Genetic Background?

Another important finding established by GWAS is that a substantial proportion of their hits are shared between several immune-mediated inflammatory diseases (IMID) (62). It has long been recognized that IMIDs cluster in both families and patients (14, 63) suggesting shared susceptibility factors between those diseases. Immunochip has greatly enhanced our understanding of such genetic overlap as the same chip was used in a large number of diseases.

The comparison of Immunochip results between 6 IMIDs (AS, coeliac disease, inflammatory bowel disease, psoriasis, rheumatoid arthritis, and type 1 diabetes) led to the identification of 71 loci significantly associated with two or more of those diseases, but not always with the same variant or in the same direction of association (i.e., protective or at risk) (64). A more sophisticated analysis was performed by Ellinghaus et al. based on Immunochip data but with a different set of diseases (AS, Crohn's disease, psoriasis, primary sclerosing cholangitis, and ulcerative colitis) (14). A significant genetic overlap was found for almost every possible pairs of 5 diseases. Additional analysis showed that this observed genetic sharing was mainly due to true pleiotropy (i.e., sharing of risk alleles by disease A and disease B) rather than heterogeneity (i.e., a subgroup of disease A cases having a higher load of risk alleles for disease B).

## Beyond the GWAS ERA: Functional Genomics of SpA

GWAS discoveries have led to a better comprehension of SpA susceptibility. It is now time for functional studies aimed at delineating the causal genetic variants and biological mechanisms underlying the genetic associations.

### Gene Expression Profiling

Genome analysis is one level of exploration of the susceptibility to complex diseases. However, it is a static view of a dynamic process and does not take into account important factors such as environmental factors. Transcriptome analysis can provide information complementary to those generated from genetic studies. To date, gene expression studies performed in SpA have led to disappointing results with no clear dysregulated pathway reproducibly identified. High throughput RNA sequencing focusing on cell types or tissues specifically involved in SpA in large samples should probably be more fruitful.

#### Microarray-Based Gene Expression Profiling: Disappointing Results

All the published gene expression profiling studies performed in SpA patients used microarrays to measure gene expression (65–77). Their results are summarized in Table 3. Each study pinpointed candidate genes or pathways. However, differentially expressed genes were most often not validated in independent cohorts and the overlap between the studies was very poor. Several reasons can explain such poor reproducibility: limited statistical power (due to small sample size), technical limitations (due to the microarray-based gene expression assessment), and biological heterogeneity of studied samples

**Table 3:** Gene expression profiling studies in spondyloarthritis.

**Study**	**Year**	**Sample size (case/control)**	**Phenotype**	**Sample type**	**Number of differentially expressed genes**	**Key findings**
Gu et al. (65)	2002	7/20	SpA	PBMC	7	Overexpression of CXCR4
Gu et al. (66)	2002	5/6	SpA	SI joint fluid	23	Overexpression of BiP compatible with the UPR hypothesis.
Rihl et al. (67)	2008	3/3	SpA	SI joint fluid	86	Overexpression of IL-7
Smith et al. (68)	2008	8/9	AS	Macrophages	141	Reverse IFN signature
Gu et al. (69)	2009	49/20	SpA/AS	PBMC	44	Overexpression of TNFα- and IL-17–inducible RGS1
Sharma et al. (70)	2009	18/25	SpA	Whole blood	1,090	Dysregulation the innate immune system and bone remodeling factors pathways
Duan et al. (71)	2010	18/18	AS	PBMC	452	“Immunosuppressive” phenotype in SpA
Assassi et al. (72)	2011	16/14	AS	Whole blood	83	Upregulation of TLR4 and TLR5.
Pimentel-Santos et al. (73)	2011	18/18	AS	Whole blood	221	Dysregulation of cartilage and bone metabolism pathways
Xu et al. (74)	2012	18/6	AS	Hip joint ligament	519	Differential gene expression in the immune system, intracellular or extracellular signaling pathway, and bone matrix biosynthesis pathway
Thomas et al. (75)	2013	8/7	SpA	Synovial biopsy	416	Altered expression profiling associated with both systemic inflammation as well as local tissue alterations
Yeremenko et al. (76)	2013	63/37	SpA	Synovial biopsy	64	Disease-specific myogenic signature
Talpin et al. (77)	2014	9/10	SpA	MDDC	81	Dysregulation of Wnt signaling pathway

SpA, spondyloarthritis; AS, ankylosing spondylitis; PBMC, peripheral blood mononuclear cells; SI, sacroiliac; MDDC, monocyte-derived dendritic cells; ND, not done; UPR? IFN?

Most of the studies were focused on peripheral blood samples, either from whole blood (70, 72, 73) or from unsorted (65, 69, 71) or isolated peripheral blood mononuclear cell populations (68, 77). It is very difficult to find a common thread between these studies, some of them suggesting a pro-inflammatory gene expression profile in SpA patients [e.g., upregulation of TLR4 and TLR5 (72)] whereas others indicating an immunosuppressive profile (71).

A limited number of studies have directly examined sites of inflammation, including sacroiliac joint fluid (66, 67), synovial biopsy (75, 76), and hip joint ligament (74). The most promising findings appear to come from synovial biopsies with the identification of a myogenic profile suggestive of fibrotic changes in the synovium of SpA patients (75, 76). An indirect comparison of gene expression between synovial tissue and PBMCs was performed by Thomas et al. (albeit samples were not obtained from the same individuals). Interestingly, immune/inflammation associated genes were found altered in the two datasets. On the other side, myogenic or oxidoreductase pathways were altered only in the synovial tissue (75).

#### Improvement Strategies

Use of high throughput RNA sequencing is very promising. Indeed, RNA sequencing carries several advantages over microarrays. It provides a broader dynamic range and also allows to detect more RNA features such as unknown genes, splice variants, and non-coding RNAs (78).

The most critical point to better understand genomic dysfunction underlying SpA pathogenesis is to study cell types or tissues known as relevant for the disease. As already discussed, most of the studies to date have concerned whole blood or peripheral blood mononuclear cells which contain a broad spectrum of cell types that may vary in proportion between individuals. As SpA is an immune-mediated disease, focusing on the immune system is logical.

To understand which cell type(s) may contribute to immune diseases, Farh et al. performed a comparative analysis between location of the GWAS-associated SNPs and epigenetic marks (79). Using available data from genome-wide studies in 39 traits including AS, they developed an algorithm to predict causal SNPs. Simultaneously, they generated chromatin maps for 56 cell types. Comparing SNP locations with chromatin maps allowed them to predict cell types contributing to each phenotype. Accordingly, the immune cells that were the most likely to contribute to AS were T cells (especially the Th17 subset) and monocytes. Interestingly, they also found enrichment in duodenal and colonic mucosa highlighting the potential involvement of the gut in addition to other more obvious sites of inflammation (including synovial tissue, enthese, cartilage, or bone).

Gene expression profiling is typically applied on samples consisting of thousands or millions of cells. However, there is growing evidence that transcriptome of even closely related cells exhibits considerable heterogeneity. Single-cell genomics is likely to significantly impact on our understanding of the functional genomics of disease (80). There is no example to date of single-cell analysis in SpA. The interest of studying very homogeneous cell populations has however been highlighted by Al-Mossawi et al. (81). Focusing on cells which produce GM-CSF, they identified a subpopulation of GM-CSF+CD4+ cells which have a specific transcriptional signature characterized by an increased level of GPR65, a proton-sensing receptor associated with SpA in GWAS.

### Follow-Up of GWAS Hits: From Variant to Function

The first step to translate GWAS hits into mechanistic understanding is to determine the causal variant(s). This step is challenging because of the high linkage disequilibrium often observed around the lead GWAS marker(s) and also because the gene(s) actually modified may lie quite distantly (up to several hundreds of kilobases) from the causative variant (82). The next step is to understand the functional consequences of the variant(s). This is also a challenging process because most disease-associated GWAS SNPs are found in non-coding regions, strongly suggesting a regulatory effect of the variants on gene expression. To overcome these issues, a current strategy consists in identifying variants that influence gene expression at various levels and in looking for an overlap of these variants with GWAS hits (83). In SpA, this endeavor is just at its beginning. The three most investigated loci outside of the MHC have been *ERAP1, IL23R*, and *RUNX3*.

#### ERAP1

Due to extensive linkage disequilibrium within and surrounding the *ERAP1* gene (including *ERAP2* and *LNPEP*), it has been difficult to identify causal variants. A 2-mutation model has been validated by conditional analysis with a primary effect due to rs30187 and a secondary effect due to two SNPs rs10050860 or rs17482078 (35). Evans et al. also demonstrated a strong gene-gene interaction between rs30187 and HLA-B27. After conditioning for the association of ERAP1, strong associations remained across the *ERAP2* and *LNPEP* genes either in HLA-B27 negative and positive patients (27, 84). However, causal variants in *ERAP2*/*LNPEP* region could not be identified genetically. IGAS Immunochip data also suggested an association of *NPEPPS* with SpA but validation of this association and identification of the causal variants at this locus will require further studies (27).

Functional consequences of several *ERAP1* variants have been determined. As the lead SNPs in this locus are non-synonymous coding variants, it has first been demonstrated that associated variants led to modifications in the three-dimensional structure of the protein (85). Another hypothesis is that *ERAP1* polymorphisms could affect gene expression level (34). This hypothesis was confirmed by several studies (86, 87) showing a strong influence of AS-associated variant on ERAP1 and ERAP2 total expression (at both RNA and protein levels) in both lymphoblastoid cell lines, monocytes, dendritic cells. Furthermore, key risk-associated *ERAP1* variants were associated with altered transcript splicing, leading to allele-dependent alternate expression of 2 distinct isoforms and significant differences in the type of ERAP1 protein produced.

Qualitative or quantitative alteration of ERAP1 enzymatic activity conferred by SpA-associated variants have significant effects on the HLA-B27 peptidome with each variants leading to specific modifications (88). Together with the strong genetic interaction between ERAP1 lead variants, rs30187, and HLA-B27, it points out to a general role of the MHC class I peptidome in SpA.

#### IL23R

The primary *IL23R* association with AS in Caucasians is with rs11209026, a missense variant. By changing the highly conserved arginine for glutamine at position 381, this variant modifies the interaction between IL-23R and its signaling partner, JAK-2 kinase, in a loss-of-function manner. Indeed, carriers of the protective allele have decreased IL-17 and IL-22 production and a lower percentage of circulating Th17 cells (56).

In addition, a second independent signal has been identified in the intergenic region downstream of IL23R and upstream of IL12RB2. In two separate studies, Roberts et al. have tried to uncover the mechanism underlying this second association signal. They identified rs11209032 as the probable causal SNP in this region. This SNP forms part of an enhancer, allelic variation of which may influence Th1-cell numbers (89). They also pinpointed a possible role for rs924080 but whether this association is independent of rs11209032 is still not clear (90).

#### RUNX3

GWAS also show compelling association between *RUNX3* variants and AS (35, 36). IGAS Immunochip study demonstrated that 22 SNPs in a 15 kb LD block upstream of the *RUNX3* promoter are strongly associated with AS with rs6600247 as the lead SNP (27). *RUNX3* encodes a transcription factor critically involved in CD8 lymphocyte differentiation and SNPs in *RUNX3* that were associated with AS also showed association with decreased CD8 positive T lymphocytes count (91).

By studying the 15 kb LD block upstream of the *RUNX3* promoter, Vecellio et al. have shown that it contains a putative regulatory region in which rs4648889 is probably the causal variant. The risk allele of this variant reduces recruitment of the interferon regulatory factor 4 (IRF4) transcription factor, leading to reduced RUNX3 expression in CD8+ T cells in an allele-dependent fashion (92). The same team also identified a second independent signal with rs4265380 which might have a regulatory impact on monocytes rather than on CD8+ T cells highlighting the cell-specificity of regulatory variants (93).

## From Bench to Bedside

One of the ultimate objectives of genetic research is to drive translational advances that enable more effective diagnosis, management, and treatment of disease.

### Diagnosis

SpA is often diagnosed late in the course of the disease with a mean delay of 8 to 10 years (94). Thus, there is a need for new diagnostic tests and genomic tools are appealing in this regard. Thomas et al. have recently tested a genetic risk score based on common variants from the Immunochip array to diagnose SpA (95). The score performed quite well to separate AS cases but had a low predictive value to identify SpA cases (including non-radiographic cases) in a cohort of chronic back pain patients. Further studies including a higher number of susceptibility variants are probably needed to obtain a more accurate diagnostic tool.

### Prognosis

Identification of genetic predictors of poor prognosis would significantly contribute to optimal treatment strategies for SpA patients. An outcome factor commonly used in genetic studies is radiographic severity, which has been shown to be partly heritable (96). Several factors of radiographic severity have been identified but often with a low level of evidence (97, 98). Additional studies, including longitudinal ones, taking into account environmental factors already known to have an impact on radiographic progression such as smoking, would be necessary to identify robust genetic predictors of structural severity.

### Treatment

Genetic studies might impact disease treatment in a number of different ways. First, as previously mentioned GWAS have uncovered previously unsuspected biological pathways that could be targeted with drugs. For example, ERAP1 inhibition could be an effective treatment in SpA. Based on the Immunochip meta-analysis of 5 IMIDs including AS, Ellinghaus et al. identified nine drug target genes which could represent promising candidates for novel drug discovery and gave the example of CCR5 antagonists which could be potential new drugs for treatment of AS (14). Studying the functional consequences of polymorphisms associated with disease can also pinpoint to pathogenic pathways as demonstrated in other complex diseases (99).

Genetics may also help to optimize treatment strategy through the identification of genome variants that influence drug efficacy and/or toxicity. In SpA, pharmacogenetics studies have focused on TNF-α inhibitors. The TNF gene is an obvious target that has already been examined as for the influence of genetic polymorphism on the response to TNF-α inhibitors. The SNP at position −308 represents the best studied marker but with conflicting results between studies (100).

The comparison of gene expression profiles before and after drug administration in relation with the level of response to treatment might also be useful to identify a gene signature predictive of treatment outcome. In 2010, Haroon et al. compared gene expression of 16 AS patients before and 2 weeks after Infliximab. They identify one molecule sLIGHT which decreased significantly after TNF blocker (101). The development of gene expression signature as a biomarker able to predict individual patient responses is more advanced in the contexte of IBD, a clinical condition related to SpA and in which TNF blockers are also used. Recently Telesco et al. tested in a phase 2a open-label study the performance of a gene expression signature identified in colon biopsies to predict efficacy of golimumab. The gene expression signature identified patients with mucosal healing with 87% sensitivity but only 34% specificity, limiting its clinical utility (102).

## Conclusion

The strong genetic background of spondyloarthritis and in particular of ankylosing spondylitis, the prototypical form of the disease, is well established. Apart from the long-standing identified strong association with HLA-B27, hypothesis-free genetic approaches have uncovered several other genetic factors involved in disease susceptibility and roughly a third of the genetic risk in AS has been explained. However, except for a few loci including ERAP1, IL23R, and RUNX3, further studies are needed to confirm and better define their involvement in the disease process.

As we enter the post-GWAS era, new challenges are arising, notably including the identification of causal variants and the characterization of their functional consequences, and a better understanding of how the variants interact together and with the environment. Addressing these issues successfully is essential to convert the genetic knowledge into clinical application.

## Author Contributions

All authors listed have made a substantial, direct and intellectual contribution to the work, and approved it for publication.

### Conflict of Interest Statement

The authors declare that the research was conducted in the absence of any commercial or financial relationships that could be construed as a potential conflict of interest.
